# Chemically-Controlled Ultrafast Photothermal Response in Plasmonic Nanostructured Assemblies

**DOI:** 10.1021/acs.jpcc.2c00364

**Published:** 2022-03-30

**Authors:** Andrea Schirato, Luca Moretti, Zhijie Yang, Andrea Mazzanti, Giulio Cerullo, Marie-Paule Pileni, Margherita Maiuri, Giuseppe Della Valle

**Affiliations:** ‡ Dipartimento di Fisica, Politecnico di Milano, Piazza Leonardo da Vinci, 32, I-20133 Milano, Italy; § Istituto Italiano di Tecnologia, via Morego 30, I-16163 Genova, Italy; ∥ Key Laboratory of Colloid and Interface Chemistry, Ministry of Education, School of Chemistry and Chemical Engineering, Shandong University, 250100 Jinan, China; ⊥ Istituto di Fotonica e Nanotecnologie - Consiglio Nazionale delle Ricerche, Piazza Leonardo da Vinci, 32, I-20133 Milano, Italy; # Department of Chemistry, Sorbonne University, 75005 Paris, France

## Abstract

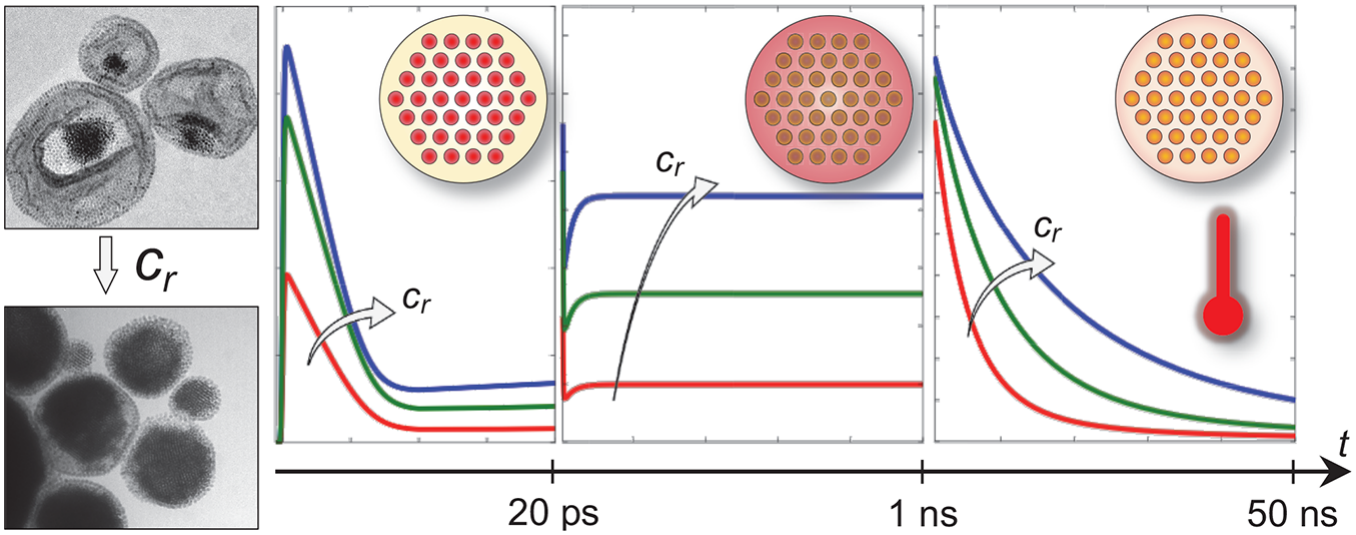

Plasmonic nanoparticles are renowned as efficient heaters due to their capability to resonantly absorb and concentrate electromagnetic radiation, trigger excitation of highly energetic (hot) carriers, and locally convert their excess energy into heat via ultrafast nonradiative relaxation processes. Furthermore, in assembly configurations (i.e., suprastructures), collective effects can even enhance the heating performance. Here, we report on the dynamics of photothermal conversion and the related nonlinear optical response from water-soluble nanoeggs consisting of a Au nanocrystal assembly trapped in a water-soluble shell of ferrite nanocrystals (also called colloidosome) of ∼250–300 nm in size. This nanoegg configuration of the plasmonic assembly enables control of the size of the gold suprastructure core by changing the Au concentration in the chemical synthesis. Different metal concentrations are analyzed by means of ultrafast pump–probe spectroscopy and semiclassical modeling of photothermal dynamics from the onset of hot-carrier photogeneration (few picosecond time scale) to the heating of the matrix ligands in the suprastructure core (hundreds of nanoseconds). Results show the possibility to design and tailor the photothermal properties of the nanoeggs by acting on the core size and indicate superior performances (both in terms of peak temperatures and thermalization speed) compared to conventional (unstructured) nanoheaters of comparable size and chemical composition.

## Introduction

The photothermal properties of plasmonic (i.e., metal-based) nanostructures and nanocomposite materials have been the subject of intensive research over the last years.^1,2^ The control of light-to-heat conversion processes at the nanoscale, both in terms of efficiency and dynamics, is indeed pivotal in a variety of fields in science and technology.^3,4^ For instance, applications of plasmon-based nanoheating range from hydrogen production^5,6^ to photocuring,^7^ from light harvesting^8,9^ and photocatalysis^10−12^ to steam generation^13−15^ and water purification.^16,17^ Similarly, in nanomedicine, molecule detection,^18,19^ together with drug delivery^20,21^ and cancer therapies,^22,23^ are further examples of techniques exploiting the photothermal dynamical response of plasmonic nanocrystals (NCs). Such a great interest in plasmonic materials is motivated by the efficient and easily tunable light-to-heat conversion upon the excitation of localized surface plasmon resonances (LSPRs),^24,25^ that is, coherent oscillations of the metal conduction electrons. The latter rapidly decay nonradiatively into a nonequilibrium distribution of hot carriers that equilibrate with the colder metal lattice,^26^ entailing the heating of the surrounding microenvironment.^27−30^ Importantly, such a mechanism occurs at an ultrafast rate and generates a strongly localized and finely controllable increase in temperature, which makes plasmonic nanostructures particularly suitable as nanosources for local heating.

In most photothermal studies and applications, ensembles of noninteracting plasmonic NCs are employed. However, in pioneering works on plasmonic heating,^31^ the most favorable configuration to heat up the environment has been shown to be in the form of NC assemblies. Indeed, as NCs aggregate, collective thermal effects take place thanks to the interaction between neighboring nanostructures, effectively promoting a substantial heating. In this collective regime, hot spots at high temperatures featuring fast dynamics can be generated in the embedding environment, outperforming isolated NCs both in the speed and magnitude of the temperature change.^31−33^ Assemblies giving rise to such a collective heating mechanism are mainly of two kinds: they can either be supported on a planar substrate or they can be in the form of free-standing aggregates. In the former case, assemblies are more often periodic arrays, that is, so-called quasi two-dimensional metamaterials.^34,35^ However, the presence of the substrate prevents such structures from being employed as real-world photothermal agents for light-driven in vivo applications. On the contrary, free-standing assemblies, also referred to as suprastructures, can be synthesized in water-soluble configurations.^36^ Interestingly, despite the theoretical predictions^31,33^ of superior nanoheating performances from plasmonic assemblies, comprehensive studies on the advantages of collective effects in suprastructures as well as applications of nanoassemblies to localized heating have been limited until very recently.^37−40^ This holds particularly true for assemblies in the free-standing configuration, the synthesis and design of which is of great interest, for example, for nanomedicine applications, such as photothermal therapies and targeted drug/cell delivery, requiring biocompatible solutions.

Toward this direction, recent advances in the chemical synthesis of suprastructures with finely controlled size, shape, and chemical composition have been reported.^41−43^ Inorganic colloidal NCs have been demonstrated to self-assemble in 3D ordered aggregates embedded in an organic polymeric matrix, allowing novel approaches to the control over photothermal effects.^44−46^ Such manufacturing techniques have thus enabled the synthesis of free-standing shells of ferrite (Fe_3_O_4_) NCs, referred to as colloidosomes, as well as supraballs (spherical assemblies of Fe_3_O_4_ NCs) and colloidosomes semifilled with NCs.^47^

In this work, we produce new supracrystalline colloidal eggs called nanoeggs. These suprastructures are colloidosomes (shells of ferrite NCs) having at their center assemblies of Au NCs. By acting on the Au NCs concentration, the size of the metallic core can be finely adjusted. The average sizes of these nanoeggs are 250–300 nm, whereas the Au NC assembly core size can increase to reach that of the whole colloidosome. A combination of femtosecond pump–probe spectroscopy and semiclassical modeling of the thermomodulational nonlinearities in Au is employed to investigate the ultrafast photothermal dynamical response of such water-soluble aggregates. Our results provide insight into the light-induced energy relaxation processes and the impact of the plasmonic assembly size on both the optical and the thermal response. By comparing the ultrafast differential transmittance of samples with different Au concentrations, we demonstrate the possibility to control the photothermal ultrafast response of such nanoeggs by tuning the size of the plasmonic suprastructure core. Moreover, by means of numerical simulations, we report a direct comparison between nanoeggs and unstructured nanoheaters under ultrashort laser pulse excitation. Both optical and thermal results demonstrate unambiguously the crucial role of the nanostructure for the engineering of the ultrafast photothermal properties of NC assemblies. Compared to standard nanoheaters with equivalent sizes and compositions, the nanoeggs achieve higher peak temperatures and faster thermalization dynamics. Our results extend current opportunities for the application of plasmonic nanoassemblies to light-driven drug release and photothermal therapy.

## Materials and Methods

### Fabrication

We synthesized inverted spinel ferrite NCs with magnetite and wurtzite traces as previously described,^48^ which we refer to as Fe_3_O_4_ NCs. They are coated with oleic acid (OA, [CH_3_-(CH_2_)_7_-CH=CH-(CH_2_)_7_-COOH]) and characterized by a 10-nm average diameter, with 8% size distribution. Au NCs coated with dodecanethiol (C_12_H_25_SH) are characterized by an average 5-nm diameter, with a 5% size distribution.^49,50^

To produce colloidosomes, shells of Fe_3_O_4_ NCs, 3 mg of Fe_3_O_4_ NCs were dispersed in a mixed solvent with 400 μL of chloroform and 8 μL of octadene (ODE, [CH_3_-(CH_2_)_5_-CH=CH_2_]). This colloidal solution was added to an aqueous solution containing 18 mg of dodecyltrimethylammonium bromide (DTAB, [C_12_H_25_N(CH_3_)_3_^+^Br^–^)]). The resulting solution was intensely agitated by a vortex for 30 s. Subsequently, 5 mL of ethylene glycol solution containing 0.4 g of polyvinylpyrrolidone (PVP, K30, *M*_w_ = 40000) was added swiftly into the emulsion and subjected to agitation with a vortex for another 30 s. The emulsion was then heated to 70 °C under a N_2_ protective atmosphere and kept at this temperature for 15 min to evaporate the inner chloroform phase. The suspension was finally allowed to cool to room temperature. The resulting NC assemblies were washed twice with water and redispersed in deionized water.

### Experimental Pump–Probe Measurements

Ultrafast pump–probe experiments were performed using an amplified Ti:sapphire laser (Coherent Libra), producing ∼100 fs pulses at 1 kHz repetition rate, 800 nm central wavelength, and 4 mJ total output energy. The 400 nm pump pulses used to excite the samples were obtained by the second harmonic generation of the laser output using a 1 mm thick β-barium borate crystal. The broadband probe pulses were instead generated by means of a white-light continuum generation process, achieved by focusing the 800 nm beams into a 2-mm thick sapphire plate, thus providing a probe spectrum spanning the 450–780 nm wavelength range. The pump pulses were focused to a spot size radius *w*_0_ = 137.5 μm (corresponding to an effective area  and the energy used per pulse was *E*_p_ = 100 nJ, thus achieving a fluence of ∼340 μJ/cm^2^. The temporally delayed probe pulses were focused on an 87.5 μm spot size and were collected after the sample by a high-speed spectrometer (Entwicklungsbuero EB Stresing) working at the full 1 kHz laser repetition rate. The measured quantity is the differential transmission of the probe pulses with and without the pump pulses as a function of the probe wavelength and pump–probe delays, Δ*T*/*T*(λ, *t*). All samples were measured at room temperature in solution in a 1-mm optical path quartz cuvette.

### Numerical Modeling

To describe the optical behavior of the samples both in static conditions and upon ultrashort laser pulse illumination, a combination of finite element-method (FEM) models and a semiclassical description of the ultrafast light-to-heat conversion dynamics has been employed. With the former we simulated either in unperturbed or photoexcited conditions the electromagnetic response of the nanoeggs, while the latter was used to determine the photoinduced dynamical changes in the optical properties of the structures. With the aim of capturing the most significant spectral and temporal features of the complex sample response while keeping the numerical tool agile, a reduced two-dimensional (2D) FEM model has been developed. Albeit in 2D, the considered geometry reproduces the in-plane nanostructured configuration of the suprastructures without an effective-medium-like description of the nanocomposite material, namely considering an assembly of Au NCs embedded in a polymeric matrix (mimicking dodecanethiol), surrounded by the ferrite colloidosome (which forms the shell of our nanoeggs), and immersed in water. Note that the reduction of the structure into a 2D (in-plane) geometry, introducing translational invariance in the third dimension, implies that NCs are treated numerically as nanowires infinitely extended in the out-of-plane direction. Despite this approximation, our model allows us to reproduce the most relevant aspects of the system and, in particular, to capture the plasmonic resonance behavior of the Au NCs as well as the photonic effects induced by the assembly configuration. Regarding the former, it is worth noticing that, contrary to the case of nanospheres, the plasmonic resonance in nanowires can be excited only when the electric field of incident light is transverse to the nanowire axis. Therefore, in our simulations we consider *p*-polarized plane waves. Of course, the polarizability and extinction cross-section of nanowires scale differently to those of nanospheres (see, e.g., ref (51) and references therein). However, for *p*-polarized light, the 2D model is capable of capturing the qualitative behavior in terms of the linear and nonlinear response of the plasmonic resonance of Au NC assemblies,^39^ yet keeping the numerical analysis relatively agile. Further comments on our modeling assumptions, together with the geometrical parameters used in the model, are provided in the Supporting Information, Section S1. This FEM model, simulating the electromagnetic interaction between the egg-like structure and light, enables us to estimate the wavelength dependent suprastructure transmittance, *T*(λ), in static conditions. Details on the implementation of the model are provided in the Supporting Information, Section S2.

To simulate the ultrafast pump–probe spectroscopy experiments, a dynamical rate-equation model has been employed. Its implementation is an extension of the well-established three-temperature model (3TM),^52^ widely used to describe photoexcitation of metallic^53^ as well as semiconducting^54^ nanostructures upon ultrashort laser pulse illumination. In this framework, the photoexcitation of hot carriers in the plasmonic material and subsequent ultrafast energy exchanges are described in terms of three internal degrees of freedom: the excess energy stored in a nonthermalized portion of out-of-equilibrium carriers, *N*, the temperature of the thermalized hot electrons, Θ_E_, and the metal lattice temperature, Θ_L_. To include a dissipation channel related to the thermal energy transfer from the metallic system to the polymeric matrix, a fourth temperature, Θ_O_, referred to the organic compound, has been introduced in the coupled rate equations. The resulting four-temperature model (4TM) reads, in agreement with previous studies,^39^ as follows:
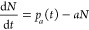
1
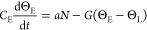
2
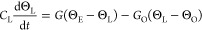
3
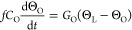
4In eq 1, *p*_*a*_(*t*) represents the electromagnetic power density absorbed by the plasmonic system following photoexcitation, while the coefficients in the four equations above govern the energy relaxation processes involved in photoexcitation. Details on definitions and values of the parameters set in the simulations are provided in the Supporting Information, Section S3. The following step in the simulation of the ultrafast response of the nanostructures starts from the temporal evolution of the energetic degrees of freedom in the hybrid plasmonic–organic system to determine the corresponding permittivity modulation photoinduced by the pump pulse absorption. To this purpose, based on the dynamical energetic variables referred to the metal (*N*, Θ_E_, Θ_L_), we followed a well-established description of Au thermomodulation nonlinearities,^53^ detailed in the Supporting Information, Section S3. In addition to the modulation of the transient permittivity in the metal, an increase in the polymer temperature ΔΘ_O_ is also able to affect the system optical properties. Indeed, a thermo-optical effect is induced in the organic matrix, resulting in a transient permittivity modulation Δε_O_. By considering the most general case of a complex-valued thermo-optical coefficient η + iζ, the resulting permittivity variation is complex as well, with a real part  and an imaginary part , with *n*_O_ representing the organic matrix refractive index.

As a final step, the FEM model detailed above is employed to determine the transient transmittance of the structure by simulating the interaction of the perturbed structure with a probe pulse impinging at a fixed delay time with respect to the pump. Numerical results are then compared to the Δ*T*/*T* signal from pump–probe spectroscopy measurements by following a perturbative approach (its implementation being detailed in the Supporting Information, Section S3).

For the system thermal response, time-dependent simulations have been performed on the same 2D geometrical domain used in electromagnetic simulations. The model implements the standard heat transfer problem in terms of the Fourier equation for heat diffusion, solved for the temperature field across the structure and considering a heat source mimicking the thermal effect of photoexcitation. Further information on expressions and parameters employed can be found in the Supporting Information, Section S4.

Lastly, regarding the comparison between suprastructures and unstructured plasmonic nanoheaters, both optical and thermal simulations have been performed for a fictive homogeneous hybrid structure, consisting of a Au nanocrystal coated with a polymeric layer, with Au and polymer content being equivalent to those considered in the corresponding assembly configuration. In the 2D model implemented, such a structure becomes a circle of radius *R*_c_, set so to match the Au volume of the 2D suprastructure model, namely , with *n*_2D_ the number of Au NCs in the 2D simulation and *r* being their radius. The obtained values roughly range between 15 and 35 nm. Except for the structure of the metallic domain, the FEM model and the dynamical optical simulations are performed following the same procedure detailed above.

## Results and Discussion

**Figure 1:**
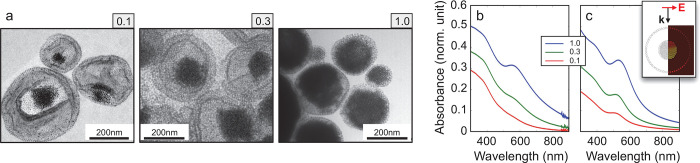
Static optical response of plasmonic suprastructures. (a) Transmission electron microscopy (TEM) images of the samples. Top labels refer to the Au concentration ratio *c*_r_, of 0.1, 0.3, and 1 from left to right, respectively. (b, c) Measured (b) and simulated (c) absorbance of the nanoeggs with *c*_r_ = 0.1 (red curves), 0.3 (green curves), and 1 (blue curves), respectively. The inset shows a schematic of the geometry used in the simulation with the corresponding normalized spatial pattern of absorption at 400 nm.

To produce colloidal “egg” structures called, for simplicity, nanoeggs, 5-nm Au NCs coated with dodecanethiol are added to the 10-nm Fe_3_O_4_ NCs dispersed in chloroform (see Materials and Methods). At the end of the procedure, “nanoeggs” are produced. Figure 1a shows that Au NCs self-assemble at the center of the colloidosomes. By keeping the same Fe_3_O_4_ concentration (3 mg) and increasing the Au concentration from 0.3 to 3 mg in 400 mL, the size of the Au NC assemblies at the center of colloidosomes progressively increases. Hence, the fixed ratio  between the concentrations (*C*) of Au and Fe_3_O_4_ (i.e., metallic core versus colloidosome), determines the size of the plasmonic suprastructure core. Here, we investigate the impact of the size of the Au NC assembly on the photothermal response of nanoeggs, with three samples corresponding to *c*_r_ = 0.1, 0.3, and 1 from left to right, respectively, in Figure 1a. The center-to-center distances between Au NCs and between Fe_3_O_4_ NCs are kept fixed (2 and 3 nm, respectively), while the Au NC assembly size undergoes substantial changes. This is clearly discernible by comparing images of the three samples, revealing that the chemically controlled size of the plasmonic core (darker spots in Figure 1a) is almost doubled between suprastructures with *c*_r_ = 0.1 (Figure 1a, left) and *c*_r_ = 1 (Figure 1a, right).

Importantly, a change in Au relative concentration has a direct impact on the static optical response of the samples, as shown in Figure 1b, which reports the measured absorbance from the three structures analyzed, namely the quantity *A* = – log_10_(*T*), with *T* being the sample transmission (see also Supporting Information, Section S1). Indeed, absolute values of absorbance increase with size over the entire visible range and the resonant feature, rather weak for the lowest concentration sample (*c*_r_ = 0.1, red curve in Figure 1b), becomes gradually more pronounced and slightly red-shifts, while the Au NC assembly size increases, resulting in a well-defined peak at ∼580 nm when *c*_r_ = 1 (blue curve, Figure 1b). The changes in the sample absorbance spectra induced by the difference in Au:Fe_3_O_4_ concentration ratio were interpreted with a newly developed model (details provided in Materials and Methods and in Supporting Information, Sections S1 and S2). Numerical results are shown in Figure 1c, where a schematic of the model is also reported (refer to panel inset). A reduced 2D geometry is defined to mimic the hybrid nanoegg configuration and excited with a monochromatic electromagnetic plane wave with in-plane polarization. The simulated absorbance spectra are in good agreement with measured data. This confirms that the spectral distortion observed when the size of the plasmonic suprastructure core is increased is ascribable to the onset of a photonic resonant mode of the Au assembly, in agreement with previous investigations.^39,41^ In these terms, the resonant feature of the plasmonic supraparticle is dominated by a collective response of the metallic NCs, rather than being dictated by the quasi-static resonance of the single NCs. As such, colloidal nanoeggs with a large core size behave as Mie-like nanoscatterers.

**Figure 2:**
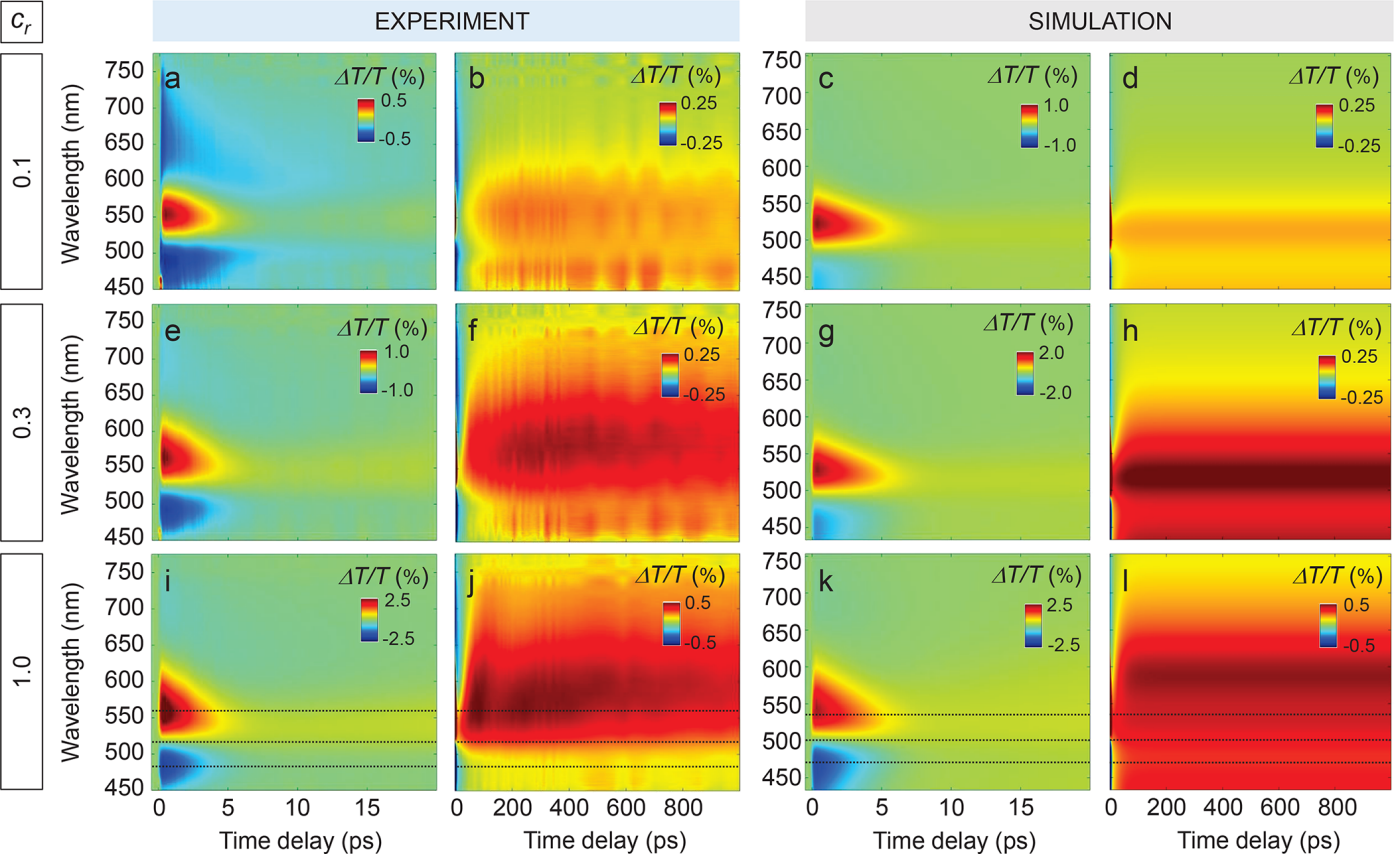
Transient optical response of plasmonic suprastructures for varying Au concentration. (a–d) Experimental (a, b) and simulated (c, d) 2D maps of the ultrafast transient differential transmittance from the suprastructure sample with 0.1 Au concentration, excited at λ = 400 nm pump wavelength and *F*_p_ ∼ 340 μJ/cm^2^ pulse fluence, shown at short (a, c) and long (b, d) pump–probe time delays. (e–h) Same as (a)–(d) for the sample with a concentration of 0.3. (i–l) Same as (a)–(d) for the 1 concentration sample.

The static behavior of nanoeggs with different values of *c*_r_ suggests that the chemical control of the Au relative concentration, resulting in a tuning of the plasmonic core size, can act as a new and efficient degree of freedom to control the static optical response of these nanoassemblies. Interestingly, this degree of freedom can be exploited to control also the photothermal dynamics of the structures. To demonstrate such a capability, we performed ultrafast pump–probe spectroscopy experiments. The samples are excited with intense (pump) laser pulses of duration ∼100 fs at λ_p_ = 400 nm wavelength. The relative differential transmittance Δ*T*/*T*(λ, *t*) experienced by a broadband weak (probe) pulse, arriving on the sample at a time delay *t* with respect to the pump pulse, is then recorded as a function of *t* and probe wavelength λ (details of the experimental procedure provided in the Materials and Methods). We also performed simulations of the nonequilibrium optical response aimed at modeling quantitatively the pump–probe experiments (see Materials and Methods and Supporting Information, Section S3 for details on the theoretical model). The main results of this combined experimental and theoretical investigation on the transient optical response of the samples are reported in Figure 2, each row corresponding to a fixed value of relative concentration *c*_r_, namely 0.1 (Figure 2a–d), 0.3 (Figure 2e–h), and 1 (Figure 2i–l), from top to bottom. Both short (up to 20 ps) and long (up to 1 ns) time scales are analyzed for a probe pulse in the visible.

By focusing on the measurements of the sample with the lowest concentration, *c*_r_ = 0.1 (Figure 2a,b), its transient response at short delays (Figure 2a) exhibits a short-lived positive lobe, centered at ∼550 nm and extinguished within ∼5 ps. Both at longer and shorter wavelengths, two negative bands arise on an ultrafast time scale as well. Such features can be attributed to the characteristic broadening and shift effects of the LSPR of the individual NCs, well reported in literature.^56,57^ On the other hand, at longer time delays (Figure 2b), the Δ*T*/*T* signal displays a peculiar dynamic, which is not commonly observed in plasmonic systems on such ultrafast time scales and was almost unexplored until very recently.^39^ Indeed, instead of decreasing monothonically toward the initial equilibrium state, the Δ*T*/*T* signal undergoes a delayed build-up. The onset of such further buildup of the signal occurs within less than 100 ps and gives rise to a long-lived broad (almost 200 nm) positive band in the Δ*T*/*T* spectrum. In agreement with previous studies,^39^ this spectral feature can be related to the ultrafast thermo-optical response from the organic matrix embedding the Au assembly. Simulations of the sample with *c*_*r*_ = 0.1 well match experiments, despite the reduced 2D model employed, which is still capable of accounting for the main features of the ultrafast optical response. At short delays (Figure 2c), the transient signal from the suprastructure is explained as a purely plasmonic fingerprint of the NC assembly. Indeed, its spectral and temporal features are reproduced by including in the model the photoinduced permittivity modulation of Au NCs, which is dictated by the ultrafast nonlinear dynamics of the internal degrees of freedom of the metal, namely the excess energy in a nonthermalizd portion of out-of-equilibrium carriers, an electronic temperature, and the Au lattice temperature (further details are in Materials and Methods and Supporting Information, Section S3). The mismatch in the red-shifted negative band of the differential transmittance, weaker in the simulations if compared to measurements, is expected to be due to the fact that the optical behavior of this sample (consisting of nanoeggs with the smallest core size) might be more sensitive to size dispersion and to the specific structural configuration within the Au assembly. The reduced 2D model, assuming identical and ordered (in a hexagonal lattice) NC aggregates, could therefore prevent the reproduction of the transient spectrum in all details. The simulated signal at longer delays (Figure 2d, to be compared with experiments on the same time scale in Figure 2b) is instead obtained by accounting for a modified permittivity of the organic matrix following a thermo-optical mechanism. The plasmonic assembly driven temperature increase in the polymer triggers a dynamical change in its optical properties, which results in a nonzero long-lived differential transmittance of the sample. The model implemented (details in Material and Methods and Supporting Information, Section S3) enables the retrieval of the main aspects of the signal, in particular its ultrafast onset and the two-sublobe structure.

Most importantly, pump–probe spectroscopy shows a significant impact of the relative Au content on the transient transmittance of the suprastructures, revealing the possibility to tailor the photothermal properties of nanoeggs by exploiting plasmonic assembly effects. The measured Δ*T*/*T* maps at short delays for the three samples with different Au content (Figure 2a,e,i) share the same plasmonic-related spectral features discussed above, which remain almost unchanged with concentration. The signal increases in absolute value when *c*_r_ passes from 0.1 to 0.3 and 1, as a consequence of the larger size of the plasmonic NC assembly, without substantial distortions in the spectrum. Conversely, major differences are recorded at longer time delays (compare Figure 2b, f, and j). In particular, the positive band governed by the thermo-optical effect from the organic matrix in the nanoegg core is enhanced and its buildup becomes faster with increasing *c*_r_. Furthermore, the spectral width of the lobe broadens and the more pronounced peak at longer wavelengths undergoes a red-shift of several tens of nm (compare panels in the second column of Figure 2). The control over the photothermal properties of the sample by the modification of *c*_r_ is also reproduced by numerical results (compare Figure 2d, h, and l). Simulated maps well correlate with measurements in both time regimes, namely when either plasmonic or thermo-optical effects govern the optical signal. The model, despite being a 2D simplified approach, is capable of accounting for all the main modifications in the differential transmittance introduced by the change in Au phase dimension within the nanoegg.

**Figure 3:**
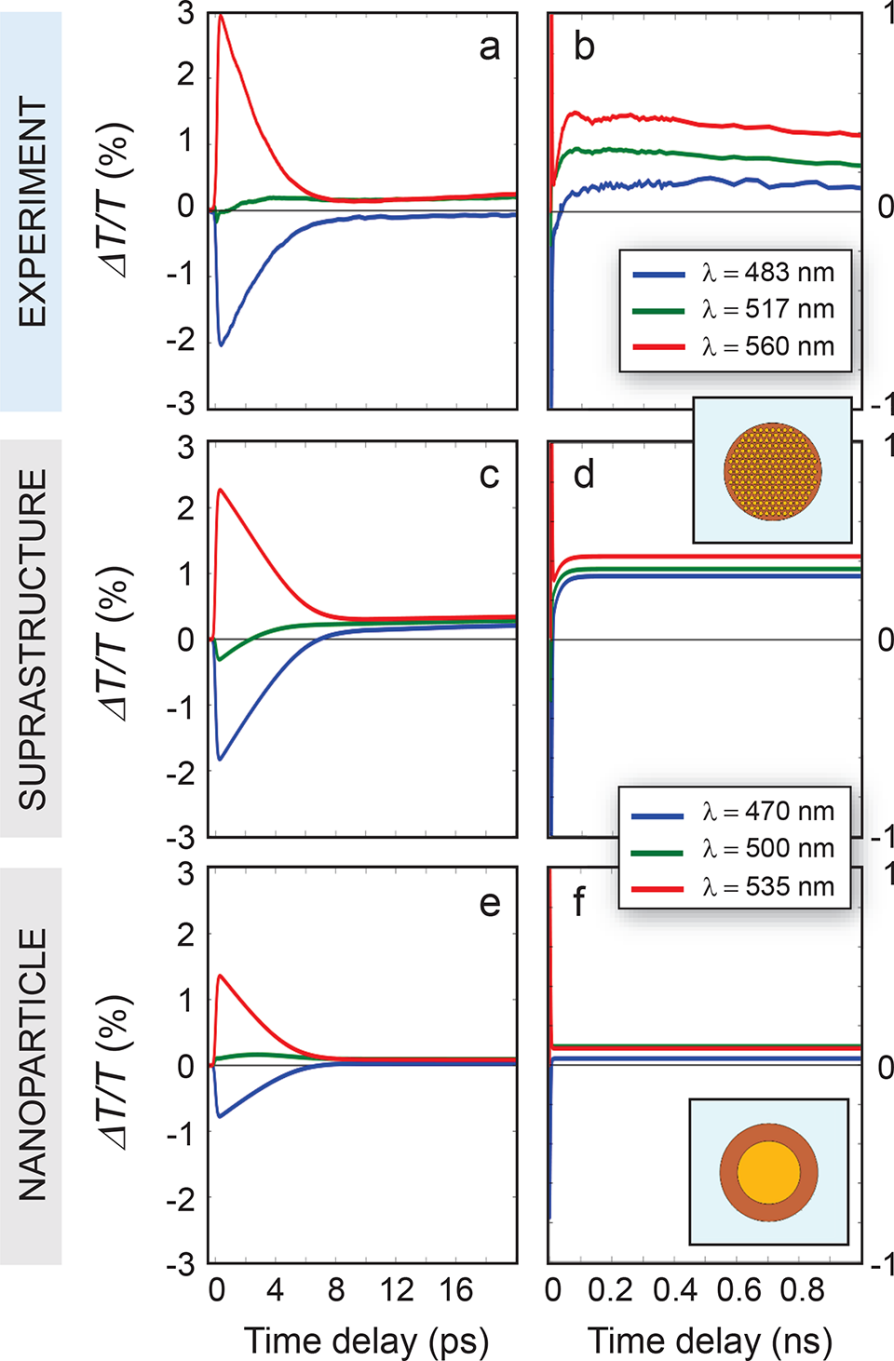
Optical dynamics of suprastructure vs coated nanoparticle. (a, b) Experimental time traces selected at specific probe wavelengths λ of the transient signal of the differential transmittance at short (a) and long (b) pump–probe time delays for the sample with Au relative concentration *c*_r_ = 1. (c, d) Simulated time sections of the optical signal at short (c) and long (d) pump–probe time delays for the same sample. (e, f) Simulated time sections of differential transmittance of a fictitious homogeneous (unstructured) system with equivalent Au volume.

To further investigate the ultrafast dynamics of the optical signal and interpret it in terms of changes in the photothermal properties of the samples, time sections of the transient transmittance are analyzed, for the *c*_r_ = 1 sample as an exemplary case. Figure 3a,b shows the measured Δ*T*/*T* for three selected probe wavelengths, corresponding to the spectral peaks of the ultrafast plasmonic lobes of Figure 2i. The signals at short (Figure 3a) and long (Figure 3b) time delays, corresponding to horizontal sections of 2D maps in Figure 2, are reported and compared to simulations (Figure 3c and d, respectively, the inset referring to the model structure). Numerical results are in good agreement with experiments, apart from a rigid shift in wavelengths. The optical signal exhibits the characteristic dynamics of photoexcited plasmonic nanostructures, with a sub-ps rise time, governed by the rate of photogeneration of thermally equilibrated hot carriers,^58^ and a decay time of a few ps, dictated by the electron–phonon scattering time. On the long time scale, time traces clearly show the signal delayed buildup pointed out when commenting the 2D maps of Figure 2. Indeed, after an ultrafast decay, the Δ*T*/*T* increases again within a few hundreds of ps, then remains positive throughout the entire time scale explored, up to 1 ns.

Most importantly, the assembly configuration of Au NCs has a pivotal role in the photothermal process of the matrix heating, which is responsible for the delayed buildup of the optical signal. Indeed, the observed behavior at longer times cannot be explained by the presence of the Au phase only, and the significant enhancement of thermo-optical effects is rather due to the nanostructuring within the suprastructure core. Hence, to ascertain the influence of collective effects from nanoassemblies on the photothermal dynamics of suprastructures, further optical simulations have been performed on a fictive unstructured system. In particular, an equivalent compact sphere (modeled as a cylinder in 2D) is considered, the volume of which is equal to the sum of the volumes of the single NCs present in the suprastructure core (details in the Materials and Methods). The obtained homogeneous nanoparticle is then embedded in the same organic matrix as the Au NC assembly, both in composition and in size. Such structure (sketched in the inset of Figure 3f) aims at mimicking more conventional colloidal nanoheaters, so to isolate the impact of nanostructuring on the photothermal properties of the water-dispersed nanoeggs. Results of the optical simulations of this fictitious structure are shown in Figure 3e and f at short and long time delays, respectively. While at short delays, no substantial differences are obtained in the predicted Δ*T*/*T* if compared to the suprastructure case (Figure 3c), a remarkable change in the signal dynamics is observed in the latter. Indeed, the differential transmittance drops and almost no optical signal is observed, despite the presence of the photoexcited metallic core, which releases heat toward the environment. In this regard, note that simulations have been performed by assuming the same scattering rate governing the heat transfer from phonons of the Au lattice to polymer phonons (the coefficient *G*_O_ in equations detailed in Materials and Methods and Supporting Information, Section S3). This is the most conservative condition for a comparison between supra- and nanoparticle configurations, which are in fact expected to exhibit different energy relaxation characteristic times. In particular, as discussed more thoroughly later on, a single NC should be associated with a slower heating rate, due to its smaller surface-to-volume ratio if compared to an assembly of NCs with the same volume of gold. In other terms, the comparison between optical signals obtained for the two (clustered and unstructured) configurations, assuming they share the same thermal properties, shows a substantially different photothermal response, which is strongly enhanced in the presence of Au nanoassemblies. Collective effects can take place, and higher temperatures are reached, enabling a more efficient heating process governing the thermo-optics observed for suprastructures.

**Figure 4:**
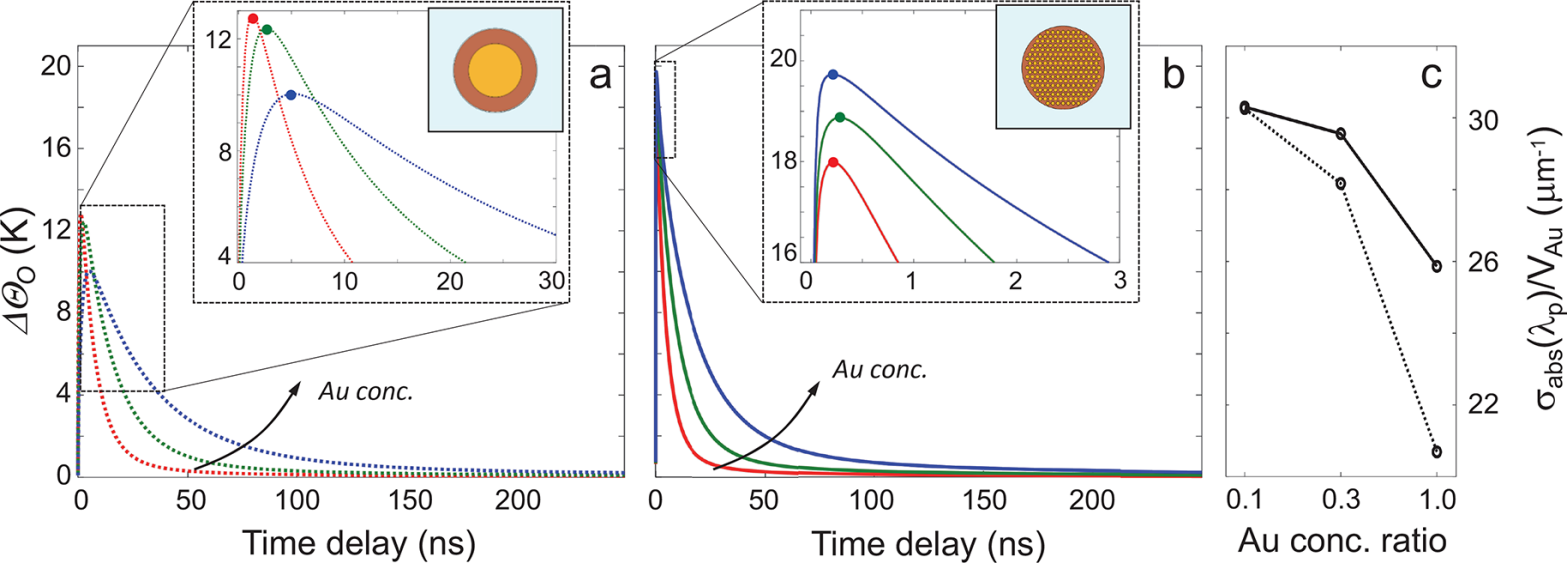
Thermal dynamics of suprastructure vs coated nanoparticles. (a, b) The average temperature increase ΔΘ_O_ in the organic coating of a conventional plasmonic nanoparticle (a) is compared with the average temperature achieved in the organic matrix of plasmonic suprastructures (b) under the same excitation conditions, for the Au:Fe_3_O_4_ relative concentration analyzed, that is, *c*_r_ = 0.1 (red curves), 0.3 (green curves), and 1 (blue curves). Colored dots highlight the maximum value of the temperature change reached. (c) Absorption cross-section normalized to the Au volume for suprastructures (solid) and corresponding coated nanoparticles (dashed). All simulations are performed in a 2D configuration.

However, the impact of nanostructuring is not limited to the ultrafast optical behavior. Most interestingly, colloidal nanoeggs enable a refined control over the dynamics of heat flow and subsequent internal thermalization of the assembly. To investigate this aspect, we performed thermal simulations upon transient heating following absorption of ultrashort laser pulses for both colloidal nanoeggs and more conventional coated NCs with the same Au concentration (see Materials and Methods and Supporting Information, Section S4 for details). The main results are shown in Figure 4.

The dynamical evolution of the average temperature increase in the organic matrix, ΔΘ_O_, is reported up to 250 ns (a time range almost 2 orders of magnitude longer than the one investigated in the transient optical measurements). This quantity is of interest as it correlates with the performances of a nanoheater: its capability to feature higher peak temperatures and higher heating and cooling rates results in both higher temperatures and temperature gradients induced in its local environment.^2^ Note that for conventional nanoparticles (Figure 4a) the cooling rate noticeably decreases with increasing amount of Au (i.e., with increasing size, for this nanoparticle configuration), thus, thermalization with the surrounding aqueous environment becomes slower. This general trend is retrieved also for nanoeggs (Figure 4b), since larger structures always exhibit larger thermal inertia, for a given configuration and chemical composition. However, the assembly configuration displays a decay time of ΔΘ_O_, which is a factor of 2 shorter with respect to the corresponding unstructured configuration for *c*_r_ = 1. Indeed, by fitting the blue dotted and solid curves in Figure 4a,b with an exponential decay function, a time constant of ∼45 ns is obtained for the former (nanoparticle), against ∼20 ns for the latter (suprastructure). An estimation of such decay times for the six structures is reported in Supporting Information, Section S5. Moreover, a key advantage from nanoeggs is provided in terms of peak temperatures, shown in the inset of Figure 4b. First of all, the rise time of ΔΘ_O_ is as fast as ∼200 ps and almost insensitive to Au concentration, its leading edge steepness being the same for the three structures (inset of Figure 4b). In fact, such feature in the dynamics of the simulated ΔΘ_O_ is confirmed experimentally by our pump–probe spectroscopy measurements, which provide a Δ*T*/*T* signal reaching a plateau with a time constant of the order of ∼200 ps for the three samples analyzed (Figure 2), giving an indirect information on the evolution of ΔΘ_O_. Indeed, in this time scale, the differential transmittance is governed by the thermo-optical response from the organic matrix embedding the Au assembly, which is proportional to the matrix temperature (see Materials and Methods). Conversely, conventional nanoheaters reach their peak temperature at longer time delays for increasing amount of Au (note that the inset in Figure 4a is extended up to 30 ns). Indeed, for larger unstructured NCs, the heat diffusion time across the structure increases, thus resulting in a slower heating dynamics. Such a mechanism, since it is governed by the surface-to-volume ratio of the nanosource of heat, only marginally affects the rise times in the case of suprastructures. In addition, note that for *c*_r_ = 1, the peak temperature is almost doubled in the assembly configuration (blue dot in the inset of Figure 4b) compared to the case of the unstructured configuration (blue dot in the inset of Figure 4a), under the same excitation conditions. Note also that in the former case the peak temperature increases (although slightly) with *c*_r_, whereas the latter case exhibits the opposite trend.

These effects can be interpreted as a direct consequence of the more efficient light-to-heat conversion process enabled by nanoassemblies. When a large number of small NCs is employed, the surface-to-volume ratio of the nanoheater becomes particularly favorable for heat exchange processes, explaining the much higher thermal performances of the assembly compared to an unstructured coated nanoparticle.

A further advantage of nanostructuring and finely controlling the metal concentration is related to the absorption of electromagnetic radiation, which in turns governs the photothermal heating process. Figure 4c shows the trend of the absorption cross-section, evaluated at the pump wavelength (λ = 400 nm) from electromagnetic simulations and normalized to the metallic volume, as a function of Au relative concentration *c*_*r*_, both for suprastructures (solid lines) and unstructured particles (dashed lines). Such a quantity directly affects the heat source driving the temperature increase across the structure [refer to Supporting Information, Section S4 and the expression for *Q*(*t*)]. Interestingly, the ratio σ_abs_/*V*_Au_ decreases with the Au concentration ratio in both suprastructures and nanoparticles, although to a lesser degree for the former. This can be understood by considering, for a fixed volume of Au, the higher penetration depth of radiation across the assembly if compared to a homogeneous configuration. In these terms, nanoassemblies enable the increase in the volume of metal with a more moderate impact on the nanoheater photothermal performances.

## Conclusions

In summary, water-dispersed colloidal nanoeggs consisting of a ferrite colloidosome surrounding a Au NC assembly embedded in a polymeric (dodecanethiol) matrix (i.e., a suprastructure) have been investigated. Different samples have been studied by modifying the Au:Fe_3_O_4_ relative concentration, *c*_r_, in the chemical synthesis, which acts as a degree of freedom to control the size of the metallic core within the nanoegg. By means of ultrafast pump–probe spectroscopy and modeling of the optical and thermal dynamical behavior of different suprastructures, we provided the evidence that their photothermal properties can be adjusted by varying the Au phase content. This holds true not only in terms of static absorbance, but also for the ultrafast transient optical response. While the plasmonic fingerprint in the differential transmittance spectra remains almost unchanged with *c*_r_, the contribution to the signal arising from the thermo-optical effect photoinduced in the organic matrix is substantially modified with Au concentration ratio. Such a mechanism is triggered by an increase of the matrix temperature (in turn, promoted by plasmonic light-to-heat conversion effects) and results in a broadband and pronounced delayed buildup of the differential transmittance optical signal within a few hundreds of ps. This in turns correlates with peculiar heat-flow dynamical features ascribable to collective effects taking place in the NCs assembly, having no counterpart in more conventional nanoheaters made of unstructured plasmonic nanoparticles. In particular, higher peak temperatures with associated faster rise times are achieved when assemblies are involved in the heating process. Also, a faster thermalization with the surrounding microenvironment is obtained, and the higher surface-to-volume ratio in suprastructures entails a more efficient heating process for a size increase if compared to unstructured systems. In a previous study,^46^ we internalized selectively either colloidosomes or supraballs, that is, solid spherical fcc assemblies of Fe_3_O_4_ NCs in tumor cells. Surprisingly, for the assemblies the Fe_3_O_4_ NCs were found to be self-assembled to the lysosome membrane. Furthermore, a marked increase of cellular uptake by tumor cells compared to dispersion of the water-soluble NC building blocks was observed. Such assemblies target different compartments of the tumor microenvironment and trigger local photothermal damages that are inaccessible for isolated NCs.^44^ Here with nanoegg suprastructures, we combine the flexibility of colloidosomes and the comparative rigidity of supraballs.^59^ Furthermore, here we observed an increase in the global temperature, different from the results obtained with colloidosomes and supraballs. From these data we can reasonably assume a very high efficiency in the photothermal effect induced by water-soluble NC assemblies, which therefore exhibit high performances as nanoheaters and could be used in several research areas and as therapeutic agents.

## Supporting Information Available

Notes S1: Reduced 2D geometry to model plasmonic nanoegg; S2: Model of the nanoegg optical response; S3: Dynamical model of nanoegg photoexcitation; S4: Model of the nanoegg thermal response; S5: Organic matrix temperature relaxation (PDF)
