# Supplementary material for: Chemically-Controlled Ultrafast Photothermal Response in Plasmonic Nanostructured Assemblies
Source: J Phys Chem C Nanomater Interfaces. 2022 Mar 30;126(14):6308–17. doi: 10.1021/acs.jpcc.2c00364 (PMC9014708; doi:10.1021/acs.jpcc.2c00364)
Supplement: Supplementary file 1 — jp2c00364_si_001.pdf [file jp2c00364_si_001.pdf]

Supporting Information for: Chemically-Controlled Ultrafast Photothermal Response in Plasmonic Nanostructured Assemblies

Andrea Schirato+,[†,‡] Luca Moretti+,[†] Zhijie Yang+,[¶] Andrea Mazzanti,[†] Giulio Cerullo,[*,†,§] Marie-Paule Pileni,[‖] Margherita Maiuri,[†,§] and Giuseppe Della Valle[*,†,§]

†*Dipartimento di Fisica - Politecnico di Milano, Piazza Leonardo da Vinci, 32, I-20133 Milano, Italy*

‡*Istituto Italiano di Tecnologia, via Morego 30, I-16163, Genova, Italy*

¶*Key Laboratory of Colloid and Interface Chemistry, Ministry of Education, School of Chemistry and Chemical Engineering, Shandong University, 250100 Jinan, China*

§*Istituto di Fotonica e Nanotecnologie - Consiglio Nazionale delle Ricerche, Piazza Leonardo da Vinci, 32, I-20133 Milano, Italy*

‖*Department of Chemistry, Sorbonne University, 75005 Paris, France*

E-mail: giulio.cerullo@polimi.it; giuseppe.dellavalle@polimi.it

S1. Reduced 2D geometry to model plasmonic nanoeggs

To simulate the complex geometry of the experimental nanoegg samples, featuring a metallic nanostructured core embedded in an organic matrix and surrounded by a ferrite colloidosome, a reduced two-dimensional (2D) model has been developed using a finite element method (FEM)-based commercial software (COMSOL Multiphysics 5.6). In this framework, Au nanocrystals (NCs) composing the plasmonic assembly have been simulated as circles (modelling spheres in 3D) of radius $r = 2.5$ nm arranged in a hexagonal lattice with an interparticle distance of 2 nm. The number n_{2D} of NCs in the assembly is then fixed by the Au:Fe_3O_4 concentration ratio and ranges between 37 and the 207 from the lowest to the highest concentration of metal. Similarly, the radius of the organic ligand matrix R_y, determining the size of the nanoegg core, is evaluated starting from the Au:Fe_3O_4 relative concentration, its estimated value varying between 25 and 55 nm. Therefore, the resulting volume of the Au phase can be estimated as $V_{Au} = N_{3D}V_i$, with $V_i = 4\pi r^3/3$ the 3D volume of the single NC, and $N_{3D} = n_{2D}R_y/r$ a rough estimation of the number of NCs in 3D, based on the one in 2D built in the FEM model geometry. Ferrite NCs in the colloidosome have instead been modelled as spheres in 2D (circles) of radius 5 nm and interparticle distance of 3 nm. The diameter of the colloidosome, ranging between 200 and 350 nm in the experimental samples, has been kept constant for all the simulated samples and set equal to 260 nm. Thus, by varying the value of the Au:Fe_3O_4 concentration ratio c_r (0.1, 0.3 or 1.0), three distinct model geometries corresponding to the considered samples are built, to be employed for both the electromagnetic and thermal FEM-based simulations (further discussed in Sections S2 and S4 respectively).

As an example, Figure S1 shows the 2D model geometry and the mesh built in the FEM-based software for the case $c_r = 1$. As noted in the main text, our reduced model describes NCs as nanowires infinitely extended in the out-of-plane direction. Although introducing a substantial geometrical difference in the modelled structure, such an approximation does not prevent us from capturing the most fundamental processes of light-matter interaction

produced by illuminating the nanoeggs, which are preserved regardless of the dimensionality of the system (as it also happens e.g. with dielectric waveguides,[1] photonic crystals[2] and metamaterials[3,4]).

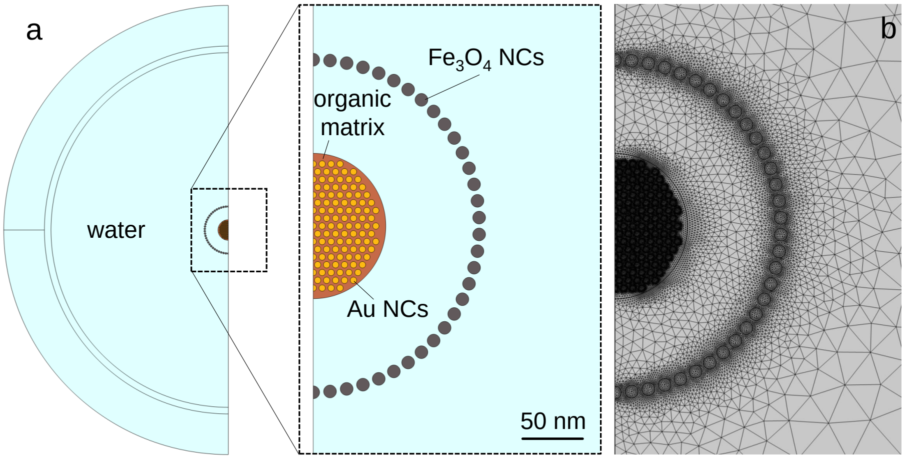

Figure S1: **Reduced 2D model geometry**. **a**, Geometrical domains defined in the numerical simulations to reproduce the optical and thermal behaviour of the plasmonic nanoegg. **b,** Mesh built across the geometrical domains for FEM-based simulations.

Indeed, the plasmonic static resonant behaviour of the samples, the ultrafast (hundreds of fs) optical modulation related to the metal electronic dynamics, and the efficient light-to-heat conversion and heating of the surrounding environment (hundreds of ps and ns) observed experimentally as a function of c_r are well reproduced in terms of the main spectral and temporal features. Likewise, the model is expected to provide reliable comparative evaluations (as the one we report on heating performances of structures, Fig. 4 from the main text) even when approximating the experimental 3D structure to its corresponding in-plane configuration.

S2. Model of the nanoegg optical response

To predict the optical behaviour of the suprastructures here investigated, a FEM-based model has been employed, utilizing the reduced 2D geometry outlined in Section S1. The numerical tool implements the electromagnetic problem in scattering formalism, and Maxwell's equa-

tions are solved in the frequency domain for an incoming monochromatic plane wave with an electric field linearly-polarised along the horizontal direction (x) and propagating along the vertical one (y). Perfectly matched layers are defined at the boundaries of the circular domain used in the simulation, so to avoid spurious effects from near-field truncation at the extrema of the geometry. The optical properties of Au have been described in terms of a complex-valued dispersed permittivity $\varepsilon_{Au}(\lambda) = \varepsilon'_{Au}(\lambda) + \mathrm{i}\varepsilon''_{Au}(\lambda)$ fitted on experimental data[5] as given in Ref. 6. In the analytical expression employed, the Drude damping factor Γ_D has been modified in order to reproduce the broadening effect in the spectral features of the optical response related to inhomogeneities and disorder in the experimental sample. A good agreement is found for a $\Gamma_D = 6\Gamma_0$, with $\Gamma_0 = 72$ meV the nominal damping coefficient in bulk gold.[6] The polymeric organic matrix has instead been treated as a dispersionless dielectric material with a purely real refractive index $n_O = 1.359$, slightly varied with respect to values reported for dodecanethiol,[7] and the refractive index of water has been set to 1.33. Finally, the optical properties of ferrite has been described in terms of a complex-valued weakly-dispersed refractive index.[8] Note that, although Fe_3O_4 exhibits a non-zero imaginary part of refractive index, its contribution to the sample absorption and corresponding dynamical modulation has been disregarded, the optical extinction of the nanoeggs in the considered wavelength range being dominated by the plasmonic response of the suprastructure core.

From the numerical solution retrieved for the scattered electric $\mathbf{E}_s$ and magnetic $\mathbf{H}_s$ fields as a function of wavelength λ, we estimated the absorption and scattering cross-section spectra, $\sigma_a(\lambda)$ and $\sigma_s(\lambda)$ respectively, of the structure.[9,10] The former is written as a 2D integral of the electromagnetic power loss density over the whole Au-NC assembly (a surface S_{Au}):

$$\sigma_a(\lambda) = \frac{\pi c \varepsilon_0 \varepsilon''_{Au}(\lambda)}{\lambda I_0} \int_{S_{Au}} |\mathbf{E}(\mathbf{r}, \lambda)|^2 \mathrm{d}S, \tag{S1}$$

where c is the speed of light in vacuum, ε_0 the vacuum permittivity, I_0 the intensity of the incident wave (in W/m, the model being in 2D), and $\mathbf{E} = \mathbf{E}_s + \mathbf{E}_0$ the total electric field, sum of the scattered and the incident ones. The scattering cross-section is similarly written

as an integral computed along a closed line Σ surrounding the scatterer:

$$\sigma_s(\lambda) = \frac{1}{2I_0}\int_\Sigma \mathrm{Re}\big[\mathbf{E}_s(\mathbf{r},\lambda)\times\mathbf{H}_s^*(\mathbf{r},\lambda)\big]\cdot\mathbf{n}\,\mathrm{d}l, \tag{S2}$$

where $\mathbf{n}$ denotes the outward pointing normal vector of the domain Σ. The 3D cross-sections of the structure have been estimated from the above 2D cross-sections as $\sigma_{3D} = \pi\sigma_{2D}R_y/2$. The transmission of the sample is then determined according to Lambert-Beer's law as $T(\lambda) = \exp\Big[-n_pN_A\sigma_eL\Big]$, with N_A the Avogadro constant, $L = 1$ mm the thickness of the cuvette sample, σ_e the (3D) extinction cross-section, sum of absorption and scattering contributions, n_p the estimated molar concentration of Au NCs. The latter has been adjusted according to the considered suprastructure and set equal to either 4.2×10^{-8} mol/m^3, 2.8×10^{-8} mol/m^3 or 1.4×10^{-8} mol/m^3 for the 0.1:1, 0.3:1, 1:1 Au:Fe_3O_4 relative concentrations. From transmission, the sample absorbance is then estimated as $A = -\log_{10}(T)$, which, according to this definition, exhibits the same spectral structure as $\sigma_e(\lambda)$, and, as the extinction, it accounts for both the processes of absorption and scattering of the incident light.

S3. Dynamical model of nanoegg photoexcitation

To model the ultrafast optical response of the plasmonic nanoassemblies constituting the metallic core, the rate-equation model introduced in the main text [refer to Eqs. (1)-(4)] and referred to as a four-temperature model (4TM) has been employed.[11] The numerical solution of the system of four coupled ordinary differential equations provides the dynamical evolution of the structure internal degrees of freedom, namely the excess energy associated to a non-thermalised portion of hot carriers in the plasmonic structures, N, together with the temperatures of metal electrons, Θ_E, metal lattice, Θ_L, and of the organic matrix Θ_O. Regarding the quantities appearing in the 4TM (Eqs. (1)-(4) of the main text), the drive

term of the photoexcitation, directly coupled to non-thermalised carriers, is $p_a(t)$, namely the electromagnetic power density absorbed by the plasmonic system, which reads:

$$p_a(t) = \sqrt{\frac{4\ln 2}{\pi}} \frac{\sigma_a(\lambda)F_p}{\tau V_{Au}} \exp\left[-4\ln 2\; t^2/\tau^2\right], \tag{S3}$$

for a pump pulse at $\lambda = 400$ nm, with fluence F_p (set to $\sim 340\ \mu\mathrm{J/cm}^2$ in the simulations) and full width at half maximum duration $\tau \simeq 170$ fs. Absorbed power density directly excites the non-thermalised carriers N, which in turn decay, for a pump at $\lambda = 400$ nm, with the hot electron heating rate[12] $a = 15$ THz. The ultrafast dynamics of electron and lattice temperatures are dictated by the heat capacity of electrons, $C_E = \gamma\Theta_E$, with $\gamma = 68\ \mathrm{J}\cdot\mathrm{K}^{-2}\cdot\mathrm{m}^{-3}$, and lattice, $C_L = 2.5\times10^6\ \mathrm{J}\cdot\mathrm{K}^{-1}\cdot\mathrm{m}^{-3}$, respectively, and $G = 2.2\times10^{16}\ \mathrm{W}\cdot\mathrm{K}^{-1}\cdot\mathrm{m}^{-3}$ the electron-phonon coupling constant. Coefficients in the equation governing the temporal evolution of the organic matrix temperature are instead $f = V_O/V_{Au}$ the ratio between volumes of polymer and Au NCs, $C_O = 0.92\times10^6\ \mathrm{J}\cdot\mathrm{K}^{-1}\cdot\mathrm{m}^{-3}$ its heat capacity per unit volume (in quantitative agreement with Refs. 11,13), $G_O = 5.8\times10^{16}\ \mathrm{W}\cdot\mathrm{K}^{-1}\cdot\mathrm{m}^{-3}$ the coupling rate between metal and matrix phonons, set as best fit on the dynamics of experimental data.

As mentioned in the main text, with the numerical solution of the 4TM at hand, providing us with the temporal dynamics of the internal degrees of freedom of the structure both for its metallic and its organic phase, the time-dependent permittivity changes photoinduced by the pump across the structure are computed. Indeed, the electronic degrees of freedom $N(t)$ and $\Theta_E(t)$ drive a modulation of the interband transitions in Au, as a result of the smearing of the distribution in energy of the out-of-equilibrium electrons. This causes a modified absorption of the impinging light, which is accounted for as a modulation of the imaginary part of the permittivity. Kramers-Kronig relations then give the corresponding real part of $\Delta\varepsilon$. The lattice temperature $\Theta_L(t)$ is instead responsible, over longer time scales, for a modulation of the Drude (intraband) contribution to Au permittivity, dictated by an increase of the electron-phonon scattering rate. The superposition of these three spectrally-dispersed

complex-valued contributions gives the total transient permittivity variation $\Delta\varepsilon_{Au}$. Further details on the physical model can be found in Ref. 12 and references therein. Note that, as for the static absorption, no modulation effects in the ferrite NCs have been included in the model. On the contrary, the complex-valued permittivity modulation $\Delta\varepsilon_O$, arising from the thermo-optical effect driven by the increase of the organic matrix temperature Θ_O and discussed in the main text has been included in the model. Regarding the parameter values set in the simulations, the complex thermo-optical coefficient $\eta+\mathrm{i}\zeta$, of the order of -10^{-4} K^{-1} for the real part and -10^3 K$^{-1}\cdot$ m^{-1} for the imaginary part within the class of polymers used,[14] has been fitted on the experimental measurements. The best-fit values considered in the simulations are in particular $\eta = -4.5\times10^{-4}$ K^{-1} and $\zeta = -2\times10^3$ K$^{-1}\cdot$ m^{-1}.

Finally, based on the dynamical evolution of the permittivity across the hybrid metallic-organic system, the transient transmittance of the structure is computed as a function of wavelength and time delay, to determine the simulated differential transmittance $\Delta T/T(\lambda,t)$, to compare with pump-probe measurements. To do so, as mentioned in the main text, a perturbative approach is implemented. In particular, the numerical relative transmittance variation is written as a linear combination of the complex permittivity modulation terms $\Delta\varepsilon_{Au}$ and $\Delta\varepsilon_O$ previously calculated. Its expression reads:

$$\frac{\Delta T}{T}(\lambda,t) = c_1(\lambda)\Delta\varepsilon'_{Au}(\lambda,t) + c_2(\lambda)\Delta\varepsilon''_{Au}(\lambda,t) + c_3\Delta\varepsilon'_O(\lambda,t) + c_4\Delta\varepsilon''_O(\lambda,t) \qquad \text{(S4)}$$

where the four $c_i(\lambda)$ functions are real-valued spectral coefficients calculated by FEM analysis. For instance, by considering $c_1(\lambda)$ (the algorithm is identical for the three others), the same electromagnetic problem discussed above is solved first in unperturbed conditions, then by applying a fictive real permittivity modification $\overline{\Delta\varepsilon'}_{Au}$, set equal to 0.1 as a good estimation of the order of magnitude of the modulation retrieved by the 4TM. The former provides then the static transmittance $T(\lambda)$, while the latter gives a perturbed transmittance

$\overline{T}(\lambda)$. The corresponding nonlinear spectral coefficient can thus be evaluated as:

$$c_1 = \frac{1}{T(\lambda)} \frac{\overline{T}(\lambda) - T(\lambda)}{\overline{\Delta\varepsilon'}_{Au}}. \tag{S5}$$

An equivalent procedure enables us to estimate the remaining coefficients and to build a 2D map of the transient broadband pump-probe spectroscopy signal to be compared with measurements.

S4. Model of the nanoegg thermal response

To estimate the temporal dynamics of the thermal response of the suprastructures, the 2D thermal model briefly discussed in the main text has been defined. Numerical simulations, performed on the reduced 2D geometry introduced in Section S1, implement the standard Fourier equation for heat diffusion: $\rho C_p \frac{\partial \Theta}{\partial t} - \kappa \nabla^2 \Theta = Q(t)$, solved for the temperature field Θ defined across the whole simulation domain (therefore $\Theta = \Theta_L$ in Au volumes, $\Theta = \Theta_O$ in the organic matrix, $\Theta = \Theta_S$ in the surrounding environment). In the equation, ρ, C_p and κ are respectively the density, heat capacity and thermal conductivity of the considered material, while $Q(t)$ is the time-dependent light-induced heat source. Its expression has been written[15] as $Q(t) = \frac{F_p \sigma_a(\lambda)}{\tau_{ep} V_{Au}} e^{-t/\tau_{ep}}$, with F_p the pump pulse fluence, σ_a is the absorption cross-section evaluated at the pump wavelength λ from electromagnetic simulation results, V_{Au} is the volume of gold, fixed by the concentration of the analysed sample. Temporal evolution of the employed heat source is governed by the electron-phonon scattering rate τ_{ep}, here set equal to 3 ps as an estimation of the characteristic time of energy transfer from electrons towards the Au lattice, which in turns becomes the heat source for the organic matrix embedding the nanoassembly, and eventually for the surrounding aqueous environment. Concerning the parameters detailing the thermal properties of materials, Au has been described[16,17] by a $\rho = 19320$ kg/m^3, $C_p = 129$ J $\cdot$ K^{-1} $\cdot$ kg^{-1}, $\kappa = 317$ W $\cdot$ K^{-1} $\cdot$ m^{-1}, while for the organic

matrix $\rho = 845$ kg/m^3, $C_p = 2187$ J $\cdot$ K^{-1} $\cdot$ kg^{-1} as in Ref. 13, and $\kappa \sim 0.04$ W $\cdot$ K^{-1} $\cdot$ m^{-1} as a reasonable estimation of the order of magnitude of thermal conductivity in similar polymers,[18] and providing consistent dynamics of the matrix temperature from optical and thermal simulations. The ferrite NCs, forming the shell of the nanoeggs, have instead been considered as passive elements (their contribution to the heating process being substantially negligible), with[19] $\rho = 4962$ kg/m^3, $C_p = 0.89$ J $\cdot$ K^{-1} $\cdot$ kg^{-1} and $\kappa = 4.9$ W $\cdot$ K^{-1} $\cdot$ m^{-1}. For water instead, $\rho = 1000$ kg/m^3, $C_p = 4186$ J $\cdot$ K^{-1} $\cdot$ kg^{-1}, $\kappa = 0.6$ W $\cdot$ K^{-1} $\cdot$ m^{-1}.[20] Thermal simulations are performed for the three different metal concentration configurations, by varying the volume of water so to keep constant the ratio between Au and water volumes. Infinite Elements (IEs) are set at the boundaries of the domain, numerically treated as infinitely extended, and boundary conditions imposing the temperature to its equilibrium value are defined beyond IEs.

S5. Organic matrix temperature relaxation

By fitting the relaxation dynamics of the organic matrix temperature increase $\Delta\Theta_O$ with a negative exponential, a characteristic decay time of the cooling rate can be extracted. Such decay time serves as a temporal estimation of the rate at which the organic matrix cools down by varying the features of the nano-heater used, i.e. suprastructure versus coated nanoparticle and by varying the Au relative concentration.

Table S1: Decay times of the temperature increase across the organic matrix (curves in Fig. 4 of the main text).

c_r	Nanoparticle	Suprastructure
0.1	10.25 ns	5.52 ns
0.3	20.86 ns	10.55 ns
1.0	43.45 ns	19.07 ns

Table S1 reports such decay times for the six scenarios detailed in Fig. 4 of the main text. Results of the fitting procedure show the advantages of using suprastructures in terms of cooling rate, as mentioned in the main text. Such decay times tend to increase with c_r in both configurations, although they remain faster by a factor of 2 when nanostructured heaters are employed.

References

(1) Burtscher, C.; Seyringer, D.; Kuzma, A.; Lucki, M. Modeling and Optimization of 1 × 32 Y-Branch Splitter for Optical Transmission Systems. *Opt. Quant. Electron.* **2017**, *49*, 396.

(2) Johnson, S.G.; Fan, S.; Villeneuve, P.R.; Joannopoulos, J.D.; Kolodziejski, L.A. Guided Modes in Photonic Crystal Slabs. *Phys. Rev. B* **1999**, *60*, 5751.

(3) Pendry, J.B.; Holden, A.J.; Robbins, D.J.; Stewart, W.J. Low Frequency Plasmons in Thin-Wire Structures. *J. Phys.: Cond. Matter* **1998**, *10*, 4785.

(4) Pendry, J.B.; Holden, A.J.; Robbins, D.J.; Stewart, W.J. Magnetism from Conductors and Enhanced Nonlinear Phenomena. *IEEE Trans. Microwave Theory Tech.* **1999**, *47*, 2075.

(5) Johnson, P. B.; Christy, R.-W. Optical Constants of the Noble Metals. *Phys. Rev. B* **1972**, *6*, 4370.

(6) Etchegoin, P. G.; Le Ru, E.; Meyer, M. An Analytic Model for the Optical Properties of Gold. *J. Chem. Phys.* **2006**, *125*, 164705.

(7) Lide, D.R.; Milne, G.W.A. *Handbook of Data on Organic Compounds,* 3rd ed. CRC Press, Inc. Boca Raton, FL, 1994.

(8) Querry, M.R. *Optical Constants*, Report No. AD-A158-623; University of Missouri, 1985.

(9) Bohren, C.F.; Hufmann D. R.; *Absorption and Scattering of Light by Small Particles*, 1st ed. John Wiley and Sons, New York, 1983.

(10) Jackson, J.D.; *Classical Electrodynamics*, 3rd ed. John Wiley and Sons, New York, 1999.

(11) Mazzanti, A.; Yang, Z.; Silva, M. G.; Yang, N.; Rizza, G; Coulon, P.-E.; Manzoni, C.; de Paula, A. M.; Cerullo, G.; Della Valle, G.; Pileni, M.-P. Light-Heat Conversion Dynamics in Highly Diversified Water-Dispersed Hydrophobic Nanocrystal Assemblies. *Proc. Natl. Acad. Sci. U.S.A.* **2019**, *116*, 8161-8166.

(12) Zavelani-Rossi, M.; Polli, D.; Kochtcheev, S.; Baudrion, A.-L.; Béal, J.; Kumar, V.; Molotokaite, E.; Marangoni, M.; Longhi, S.; Cerullo, G.; Adam, P.M.; Della Valle, G. Transient Optical Response of a Single Gold Nanoantenna: the Role of Plasmon Detuning. *ACS Photon.* **2015**, *2*, 521-529.

(13) Tutubalina, V.P.; Gabdrakhmanov, F.G.; Konyukhova, T.M. *Study of the Heat Capacity of n-Sulfides*, Teplo-Massoobmen Khim. Tekhnol., 1982.

(14) Diemeer, M.B.J. Polymeric Thermo-Optic Space Switches for Optical Communications. *Opt. Mater.* **1998**, *9*, 192-200.

(15) Baffou, G.; Rigneault, H. Femtosecond-Pulsed Optical Heating of Gold Nanoparticles. *Phys. Rev. B: Condens. Matter Mater. Phys.* **2011**, *84*, 035145.

(16) Ahscroft, N.W.; Mermin, N.D.; *Solid State Physics*, Saunders College, Philadelphia, 1960.

(17) Lide, D.R. *CRC Handbook of Chemistry and Physics*, 74th ed. Boca Raton, 1993.

(18) Schechtell, E.; Yan, Y.; Xu, X.; Cang, Y.; Tremel, W.; Wang, Z.; Li, B.; Fytas, G. Elastic Modulus and Thermal Conductivity of Thiolene/TiO_2 Nanocomposites. *J. Phys. Chem. C* **2017**, *121*, 25568-25575.

(19) Horia, F.; Easawi, K.; Khalil, R.; Abdallah, S.; El-Mansy, M.; Negm, S. Optical and Thermophysical Characterization of Fe_3O_4 Nanoparticle. *IOP Conf. Series: Mat. Sci. Eng.* **2020**, *956*, 012016.

(20) Touloukian, Y.S.; Liley, P.E.; Saxena, S.C. *Thermophysical Properties of Matter - the TPRC Data Series. Volume 3. Thermal Conductivity - Nonmetallic Liquids and Gases.* Def. Tech. Inf. Cent. 1970.
